# Volatile working memory representations crystallize with practice

**DOI:** 10.1038/s41586-024-07425-w

**Published:** 2024-05-15

**Authors:** Arash Bellafard, Ghazal Namvar, Jonathan C. Kao, Alipasha Vaziri, Peyman Golshani

**Affiliations:** 1 Department of Neurology, David Geffen School of Medicine, University of California, Los Angeles, CA USA; 2 Department of Electrical and Computer Engineering, Henry Samueli School of Engineering, University of California, Los Angeles, CA USA; 3 Laboratory of Neurotechnology and Biophysics, The Rockefeller University, New York, NY USA; 4 The Kavli Neural Systems Institute, The Rockefeller University, New York, NY USA; 5 Greater Los Angeles VA Medical Center, Los Angeles, CA USA; 6 Semel Institute for Neuroscience and Human Behavior, University of California, Los Angeles, CA USA; 7 Integrative Center for Learning and Memory, University of California, Los Angeles, CA USA; 8 Intellectual and Developmental Disability Research Center, University of California, Los Angeles, CA USA

**Keywords:** Neural circuits, Working memory

## Abstract

Working memory, the process through which information is transiently maintained and manipulated over a brief period, is essential for most cognitive functions^1–4^. However, the mechanisms underlying the generation and evolution of working-memory neuronal representations at the population level over long timescales remain unclear. Here, to identify these mechanisms, we trained head-fixed mice to perform an olfactory delayed-association task in which the mice made decisions depending on the sequential identity of two odours separated by a 5 s delay. Optogenetic inhibition of secondary motor neurons during the late-delay and choice epochs strongly impaired the task performance of the mice. Mesoscopic calcium imaging of large neuronal populations of the secondary motor cortex (M2), retrosplenial cortex (RSA) and primary motor cortex (M1) showed that many late-delay-epoch-selective neurons emerged in M2 as the mice learned the task. Working-memory late-delay decoding accuracy substantially improved in the M2, but not in the M1 or RSA, as the mice became experts. During the early expert phase, working-memory representations during the late-delay epoch drifted across days, while the stimulus and choice representations stabilized. In contrast to single-plane layer 2/3 (L2/3) imaging, simultaneous volumetric calcium imaging of up to 73,307 M2 neurons, which included superficial L5 neurons, also revealed stabilization of late-delay working-memory representations with continued practice. Thus, delay- and choice-related activities that are essential for working-memory performance drift during learning and stabilize only after several days of expert performance.

## Abstract (web summary)

Delay- and choice-related activities that are essential for working-memory performance drift during learning and stabilize only after several days of expert performance.

## Main

Working memory (WM)—the ability to temporarily store and manipulate information—is essential for most cognitive functions^1–4^ and is impaired in several neurological and psychiatric disorders^5–7^. The maintenance of information in WM is thought to be mediated by persistent, sequential or oscillatory activity^8–10^, and its representation in the state space of dynamical systems is often modelled as discrete or continuous attractors^11–15^. The mechanism of generation and maintenance of WM-related neural representations during learning and their evolution with continued expert performance remains unclear. A key challenge has been to record and manipulate the same neuronal populations over a long period of time while the animal learns and becomes an expert in the task. Here we pose two fundamental questions concerning the stability and causality of WM representations, examining (1) the stability of WM representations across time as the mouse learns the task and attains expertise through practice; and (2) the role of these representations in driving task performance.

**Fig. 1:**
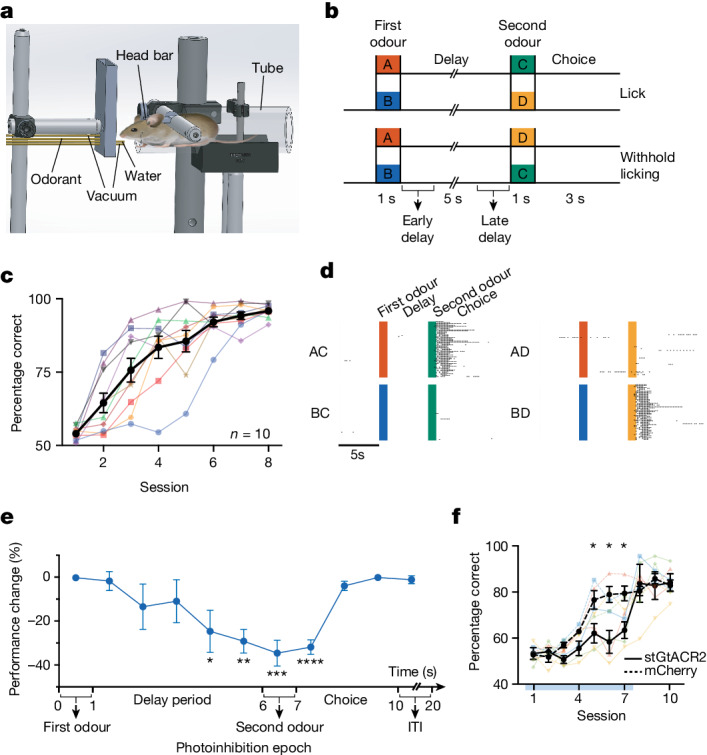
The effects of optogenetic inhibition on WM task performance. **a**, The experimental set-up. **b**, The delayed-association WM task trial types; licking was assessed during the 3 s choice period, with early- and late-delays periods noted. **c**, Learning progress across eight sessions, measured on the basis of the percentage of correct responses. **d**, Learning session example, with licks marked. **e**, Photoinhibition effect during different task epochs on the animal’s performance (fourth second of the delay period, *P* = 0.009; fifth second of the delay period, *P* = 0.005; second odour, *P* = 0.0004; first second of choice epoch, *P* = 0.0001). Statistical analysis was performed using paired *t*-tests. **f**, Photoinhibition of the M2 during the last 2 s of the delay period across the first 7 days of training impairs task performance. *n* = 4 (stGtACR2-expressing) and *n* = 4 (mCherry-expressing) mice. The *P* values determined using two-sample *t*-tests for sessions 1–10 were as follows: *P*_1_ = 0.8425, *P*_2_ = 0.4610, *P*_3_ = 0.6904, *P*_4_ = 0.0724, *P*_5_ = 0.0463, *P*_6_ = 0.0146, *P*_7_ = 0.0161, *P*_8_ = 0.7065, *P*_9_ = 0.6530 and *P*_10_ = 0.7955. For **c**, **e** and **f**, data are mean ± s.e.m. NS, not significant; *P ≤ 0.05, **P ≤ 0.01, ***P ≤ 0.001, ****P ≤ 0.0001. Details of the statistical analyses are provided in the Methods. Source Data

**Extended Data Fig. 1:**
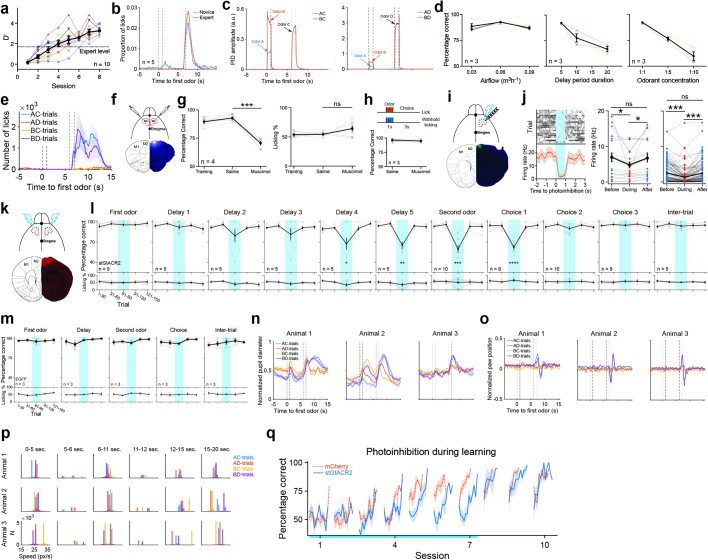
Behavioural and physiological responses in mice undergoing task learning and optogenetic manipulation. (a) Performance of the animals while they learned the task over eight days assessed with discriminability index (D’). (b) Normalized licking distribution of novice and expert mice during the 20 second trial period. (c) Odour intensity measured by photoionization detector (PID). (d) Effect of airflow (left), delay period duration (middle), and odour concentration (right) on animal’s task performance. (e) Licking distribution for different trial types in expert animals. (f) Schematic from Paxinos and Franklin^44^ illustrating the muscimol injection target and a histological section after the fluorescent dye (DiI) injection marking the injection location. (g) Muscimol’s impact on expert mice’s performance and licking, indicating significant performance disruption (paired *t*-test; 95% CI; performance *p* = 0.0001; licking *p* = 0.159). Mice received muscimol or saline randomly across consecutive sessions. Each colour indicates one mouse with two mice being experimented with twice. (h) Schematics and effect of muscimol inhibition on mice in a non-working memory task. (i) Schematic from Paxinos and Franklin^44^ illustrating the injection target and a histological section after the stGtACR2 showing injection localization. (j) Blue light illumination silences stGtACR2-expressing neurons, as demonstrated by a neuron’s activity raster plot and its averaged firing rates during photostimulation, alongside the collective activity of 20 recorded units before, during, and after inhibition, showing significant changes (paired *t*-test; before-during *p* = 0.026; during-after *p* = 0.020; before-after *p* = 0.235). The right panel is another example of the effect of blue light on modulating the activity of stGtACR2-expressing neurons showing the activity of 102 electrophysiologically recorded units before, during, and after photoinhibition (paired *t*-test; before-during *p* = 1.28 × 10^−7^; during-after *p* = 1.01 × 10^−6^; before-after *p* = 0.70). (k) Illustration of bilateral optogenetic setup and histology showing stGtACR2-expressing neurons. (l) Optogenetic inhibition during different task epochs reduces expert mice’s task performance (paired *t*-tests: delay 4, *p* = 0.0088; delay 5, *p* = 0.005; second odour, *p* = 0.0004; choice 1, *p* < 0.0001). (m) Effect of blue light on the performance (top) and licking (bottom) of expert mice expressing EGFP. The plot titles indicate the epochs at which the illumination was done. Each colour indicates one mouse. (n) Normalized pupil diameter for various trial types in three distinct animals exhibiting expert-level performance. (o) Normalized paw position across different trial types in three distinct animals demonstrating expert-level performance. (p) Distribution of forelimb speed in three individual expert animals across various odour combinations and temporal segments. (q) Photoinhibition of the M2 during the last 2 seconds of the delay period across the first 7 days of training impairs task performance. Performances are calculated over twenty-trial blocks in each session (n = 4 stGtACR2-expressing mice; n = 4 mCherry-expressing mice). (a), (b), (d), (e), (g)–(j), (l)–(o), and (q) Mean ± s.e.m. *p≤ 0.05, **p≤ 0.01, ***p≤ 0.001, ****p≤ 0.0001, ns, not significant. See Methods for details of statistical analyses. Source Data

To address these questions, we trained mice to perform an olfactory delayed-association WM task^16^ (Fig. 1a,b). In this task, water-deprived head-fixed mice were presented with odours A or B for 1 s. After a 5 s delay period, the mice were presented with odours C or D for another second. If odour C followed odour A or odour D followed odour B, the mice were rewarded with water after licking during the 3 s choice period after the second odour (Fig. 1b). For the other odour combinations, the animals learned to withhold licking. The mice were not punished for false alarms or for licking during the delay period, but their licking patterns after learning showed that licking was restricted to the choice period (Fig. 1d and Extended Data Fig. 1b). The mice learned to perform the task after training for around seven sessions, with an accuracy level of 94.2 ± 1.3% (discriminability index, *D*′ > 3) (Fig. 1c and Extended Data Fig. 1a). The performance was assessed as the ratio of hits and correct rejections over the total number of trials that the mouse completed during the training session. Several behavioural control experiments and photoionization detector measurements demonstrated that odour residues did not linger during the delay period (Extended Data Fig. 1c). Moreover, airflow modulation did not affect the animal’s performance. However, performance decreased with increasing duration of the delay period or decreasing levels of odorant concentration, as expected (Extended Data Fig. 1d).

To ensure that different odours or odour combinations result in similar behavioural responses, we measured the lick time distributions while the mice performed the task at an expert level (performance ≥ 80%). Lick time distributions for AC versus BD trials, as well as AD versus BC trials, showed no statistically significant differences (Extended Data Fig. 1e). This indicates consistent behavioural responses across different odour combinations. We also measured pupil diameter changes to distinct odour combinations and found highly consistent pupillary responses within individual animals. Pupillary responses were similar across animals for odour combinations associated with rewards (AC and BD trials) and those without rewards (AD and BC trials) (Extended Data Fig. 1n). To ensure that the neural activity that we measured in the next section was not simply a correlate of motor movements, we recorded paw movement during the delay period for different odour combinations and found no significant differences in paw movements for different trial types, suggesting that delay-related activity does not simply correlate with motor movements (Extended Data Fig. 1o). Furthermore, we measured forelimb speed distribution across odour combinations and different task epochs. Partitioning each trial into six intervals revealed marked heterogeneity in forelimb movement speed among the examined animals, indicating that different odours do not differentially and consistently impact motor behaviour (Extended Data Fig. 1p).

## Importance of late-delay epoch activity pattern

To assess the role of M2 neurons in the performance of the olfactory delayed-association WM task, expert mice received bilateral injections of either saline or muscimol into the M2 1 h before testing. M2-localized (Extended Data Fig. 1f) muscimol injections led to a performance drop (70.5% ± 1.8% versus 92.8% ± 1.2% with saline; Extended Data Fig. 1g). Licking behaviour remained unaffected by muscimol (Extended Data Fig. 1g). The performance on a non-WM task was not impaired by muscimol (Extended Data Fig. 1h), suggesting the specific importance of the M2 for WM task performance, but not simple decision-making.

We investigated the impact of inhibiting CaMKII^+^ neurons in the M2 at different task stages using soma-targeted anion channelrhodopsin (stGtACR2), which effectively suppressed neuronal firing without rebound activity (Extended Data Fig. 1i,j). Inhibition of M2 neurons only during the fourth and the fifth second of the delay period, during the second odour, and during early-choice epochs significantly degraded performance by 24.7 ± 9.6% (*n* = 5), 29.2 ± 5.4% (*n* = 5), 34.6 ± 5.9% (*n* = 10) and 31.9 ± 3.3% (*n* = 8), respectively (Fig. 1e and Extended Data Fig. 1l). By contrast, inactivation at other times did not affect performance. Illumination of the M2 in control mice injected with AAV-CaMKIIa-eGFP did not affect performance. Thus, neuronal activity in late-delay but not early-delay periods is essential for performance of the WM task.

We next examined whether suppression of late-delay activity in the M2 impedes learning of the WM task. To answer this question, we inhibited the M2 activity of mice during the last 2 s of the delay period for every trial while the animal learned the task for the first seven sessions. M2 inhibition significantly impaired performance during learning sessions in comparison to the control mice that were injected with AAV-CaMKIIa-mCherry (Fig. 1f). However, when we stopped the photoinhibition during sessions 8 to 10, the performance of the mice rapidly converged to the level of performance of the control mice, suggesting that learning had proceeded despite the deficient performance of the animal in the previous sessions (Fig. 1f and Extended Data Fig. 1q), consistent with ‘silent learning’^17^. In all instances, we histologically verified the injection site, confirming its localization to the M2 region. In no case was the M1 labelled (Extended Data Fig. 1k).

Thus, M2 neuronal late-delay and choice-period activity causally drives WM performance but, while sufficient, it is not necessary for learning of the task.

## The M2 contains task-related selective neurons

**Fig. 2:**
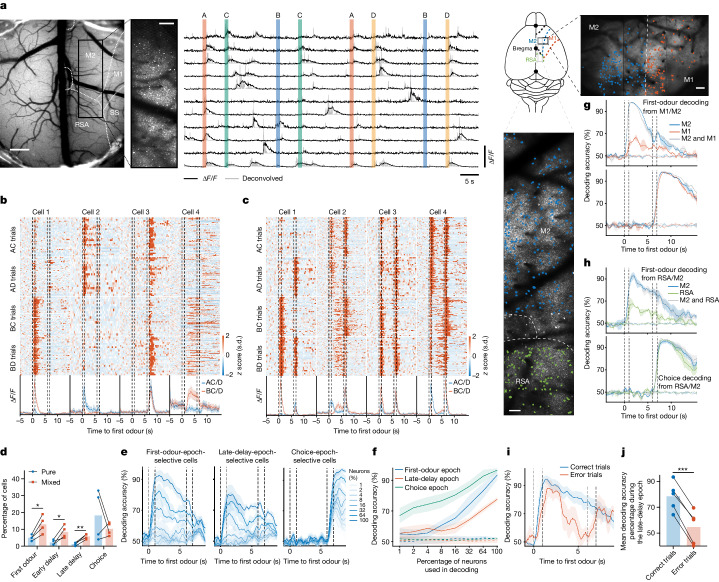
Evolution of late-delay M2 activity patterns carrying WM information. **a**, Imaging set-up and calcium activity of representative neurons across trials from one of the animals. Images of the other mice looked similar. Scale bars, 500 μm (left), 200 μm (left inset) and 100 μm (top and bottom right). ∆*F/F*, fluorescence signal change; SS, somatosensory. **b**, *z*-scored activity of four example neurons selectively responsive to specific odours, choices or delay epochs. **c**, Example neurons exhibiting mixed selectivity. In **b** and **c**, cells were sampled from at least 2 days after the animal achieved expert-level performance. **d**, The percentage of neurons exhibiting pure or mixed selective activity (*n* = 4 expert mice; first odour, *P* = 0.022; early delay, *P* = 0.035; late delay, *P* = 0.006; choice, *P* = 0.353). Statistical analysis was performed using paired *t*-tests. **e**, The accuracy of decoding the first odour (left and middle) or choice (right) using *N*% of the most selective neurons. *n* = 5 mice performing at expert level. **f**, The maximum decoding accuracy compared with shuffled data. **g**,**h**, The decoding accuracy of predicting the first odour and choice from the activity of M1 and M2 (**g**) and RSA and M2 (**h**) neurons separately and combined. The same number of neurons was used to ensure a fair comparison. **i**,**j**, Decoding accuracy comparison between correct and error trials in expert mice (**i**; *n* = 5), demonstrating significant predictive value during the late-delay epoch (*P* = 0.0001, paired *t*-test). The analysis in **g**–**j** is based on data from the last imaging day. For **e**, **f** and **i**, data are mean ± s.e.m. Details of the statistical analyses are provided in the Methods. Source Data

We recorded neuronal activity in M2 L2/3 using mesoscopic two-photon calcium imaging while C57BL/6JTg (Thy1-GCaMP6s) GP4.12Dkim/J mice performed the task at expert level (Fig. 2a). We typically recorded 622 ± 295 neurons simultaneously during each session. Within each day, we observed selective and reliable responses to the first odour during the first-odour, early-delay, late-delay and choice epochs (Fig. 2b,c). A total of 46.5 ± 4.5% of cells showed selectivity during at least one of the epochs. We quantified the selectivity of individual cells by comparing the distribution of their calcium activity based on the identity of the first odour presented at different time intervals. Neurons with selective activity during a single epoch were rare. In expert mice, 5.0 ± 0.8%, 2.3 ± 0.8%, 1.3 ± 0.5% and 18.3 ± 7.4% (*n* = 4 mice) of neurons showed pure selectivity during first-odour, early-delay, late-delay and choice epochs (Fig. 2b,d). By contrast, a larger proportion of neurons (13.0 ± 2.6%, 8.5 ± 1.9%, 5.8 ± 0.8 and 10.3 ± 1.9%) showed mixed selectivity^18^ and had strong activity during multiple task epochs (Fig. 2c,d).

**Extended Data Fig. 2:**
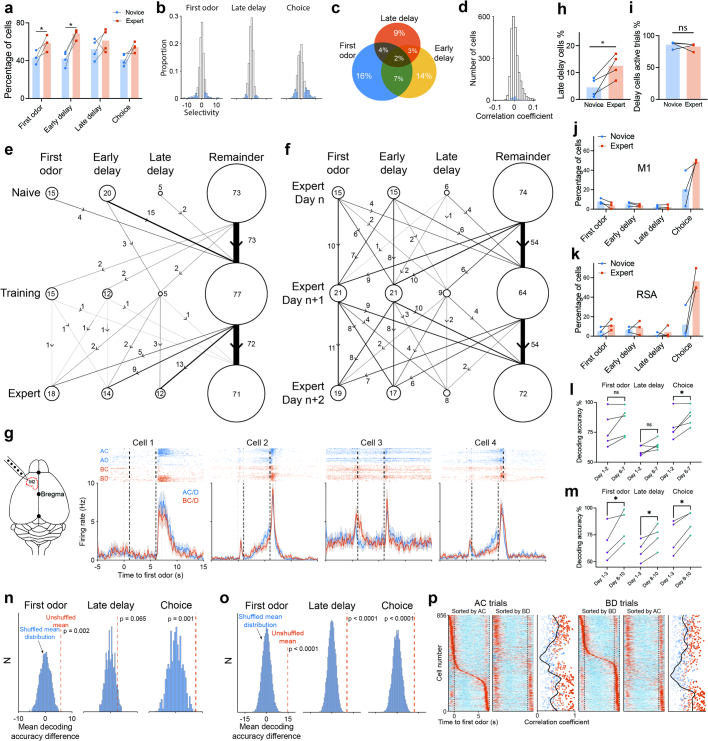
Neural selectivity and activity dynamics in mice performing a delayed-association WM task. (a) Percentage of cells with significant activity field during different task epochs. (n = 4 mice; paired *t*-tests; first odour, *p* = 0.050; early-delay, *p* = 0.011; late-delay, *p* = 0.351; choice, *p* = 0.085). (b) Selectivity distribution during different task epochs. Shades denote the significantly selective cells. (c) Percentage of selective cells overlapping during different task epochs. (d) Distribution of correlation coefficient between animal’s body movement and calcium activity of 2611 neurons (n = 5 expert mice) with mean correlation value of 0.007 ± 0.003. (e) Evolution of selective cells during different task epochs over different learning stages. The numbers denote percentages. Radius of the circles and the line thicknesses are properly scaled. (f) Similar to (e) but for expert animals over three consecutive days. (g) Electrophysiology recording of four example M2 neurons. (h) Proportion of late-delay neurons that are active in novice and expert animals (paired *t*-test). (i) Percentage of trials with active delay cells in novice and expert animals (paired *t*-test). Note that novice animals exhibit a significantly lower proportion of delay cell activity compared to experts as shown in (h). (j) Percentages of selective neurons during the first-odour (*p* = 0.11), early-delay (*p* = 0.2), late-delay (*p* = 0.51), and choice (*p* = 0.13) epochs in M1 among novice and expert mice, with statistical analyses conducted using paired *t*-tests. (k) Proportions of selective neurons in the first-odour (*p* = 0.18), early-delay (*p* = 0.63), late-delay (*p* = 0.59), and choice (*p* = 0.09) epochs within the RSA of both novice and expert mice. Statistical analyses were conducted using paired *t*-tests. (l) Average across-day decoding accuracy for individual animals in the early (first two days) and late (last two days) expert phase of task performance (paired *t*-tests) in the first-odour (*p* = 0.11), late-delay (*p* = 0.06), and choice (*p* = 0.04) epochs, extracted from Fig. 3i. (m) Similar to (l), but with corresponding p-values of 0.05, 0.03, and 0.02 for the first-odour, late-delay, and choice epochs, respectively, extracted from Fig. 4g. (n) Distribution of difference in the mean decoding accuracies when shuffling day identities. The red dashed line represents the mean change in decoding accuracy between the last three and first three days without shuffling. The p-value (*z*-test) suggests a significant difference in decoding accuracy for the first-odour and choice epochs but not for the late-delay epoch. This indicates that representations for the first-odour and choice epochs stabilize, while those for the late delay appear to drift daily. (o) Similar to (n), but using LBM data and the difference in decoding accuracies between the first and last five days. The p-value indicates a significant difference in decoding accuracy for all three epochs, suggesting that representations for the late-delay epoch also stabilize as mice continue practicing the task. (p) Z-scored activity of neurons exhibiting selectivity for A and C or B and D, sorted by their activity during AC and BD trials, and vice versa. The right panel illustrates the correlation coefficient of the neurons’ activity when sorted by AC or BD trials. Neuron activity was temporally shuffled 10,000 times to determine the significance of actual correlations against the distribution. Red markers indicate the 95th percentile. *p≤ 0.05, **p≤ 0.01, ***p≤ 0.001, ****p≤ 0.0001, ns, not significant. See Methods for details of statistical analyses. Source Data

We also quantified the proportion of trials with active delay cells in well-trained mice (Extended Data Fig. 2h,i). In novice mice (performance < 65%) on the first training day, delay cells were active in 85.8 ± 2.6% of trials. On the last recording day for expert animals, delay cells were active in 82.8 ± 2.9% of trials, indicating little change in the reliability of the responses with learning. Notably, the number of active cells during the delay period was significantly lower in novice mice (Extended Data Fig. 2h).

To ensure that the slow decay of calcium transients did not artificially give the appearance of delay-related activity, we performed single-unit electrophysiological recordings in expert animals as they performed the WM task. Consistent with our imaging experiment, these recordings showed odour-selective activity during the odour period and odour-selective ramping activity during the late-delay period (Extended Data Fig. 2g). Thus, delay-related activity recorded with calcium imaging was not an artifact of the decay of calcium transients.

The neural activity patterns recorded with calcium imaging did not correlate with the movements of the mice. From 2,611 recorded cells in five mice, only 32 cells (1.2 ± 0.2%) showed a significant correlation with the limb movements of the mice (Methods and Extended Data Fig. 2d).

## Late-delay M2 activity contains WM information

**Extended Data Fig. 3:**
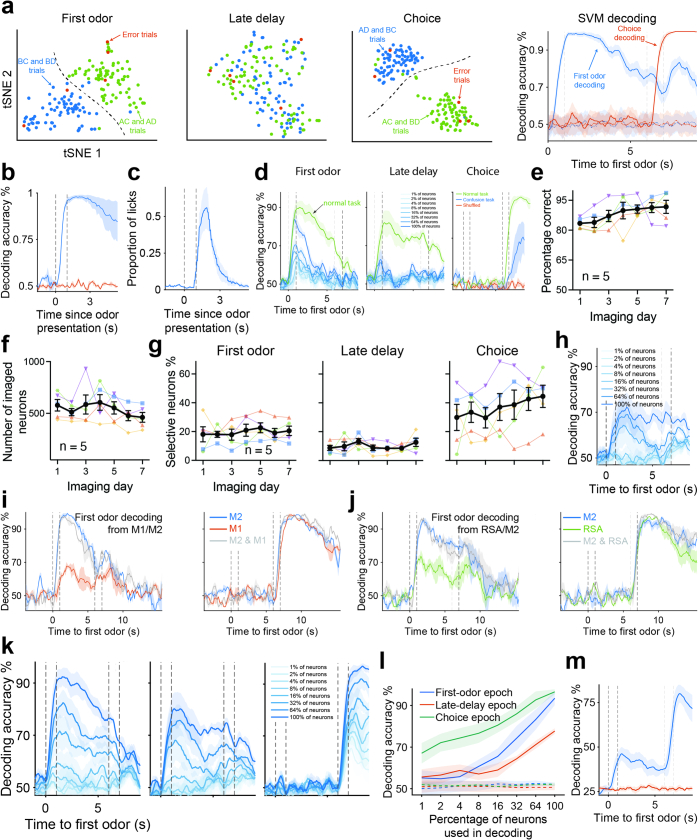
Decoding neuronal representations of the first-odour identity and choice across different task epochs. (a) Visualization of the first odour identity and choice during different task epochs using t-distributed stochastic neighbour embedding (t-SNE), in an expert animal performing at 92%. The decoding accuracy of the SVM decoder, presented in the right panel, illustrates the presence of working memory information in the neuronal population activity during the late-delay epoch for the same dataset. (b) Decoding accuracy for odour identification in the go/no-go task, devoid of a working memory component (n = 3 mice). (c) Distribution of the animal’s licking behaviour during the go/no-go task (n = 3 mice). (d) Left panel: Decoding accuracy for the first odour in normal and confusion tasks with randomized reward contingencies, using N% of the top selective neurons during first-odour and late-delay epochs. Notably, in the confusion task, decoding accuracy during the late-delay epoch approaches chance level (n = 3 mice). Right panel: Evaluation of choice decoding in both normal and confusion tasks using the activity of all neurons. Shuffled data pertains to the confusion task. (e) Behavioural performance of mice at expert proficiency levels, featuring the identical mice analysed in Fig. 3g-j (*p* = 0.25; one-way ANOVA). (f) Number of imaged neurons for each imaging day, showcasing the same mice examined in Fig. 3g-j (*p* = 0.25; one-way ANOVA). (g) Percentage of neurons exhibiting selectivity in each time epoch on every imaging day, featuring the identical mice analysed in Fig. 3g-j (first-odour epoch *p* = 0.69, late-delay epoch *p* = 0.46, choice epoch *p* = 0.34; one-way ANOVA). (h) Decoding accuracy for the first odour using N% of choice epoch’s most selective neurons. (i) Decoding accuracy in predicting the initial odour identity and choice based on the separate and combined activity of M1 and M2 neurons using a non-linear Long Short-term Memory (LSTM) recurrent neural network decoder. Equal numbers of M1 and M2 neurons are employed in the decoding process to ensure a fair comparison. (j) Similar to (i), but using simultaneous recordings of the retrosplenial and secondary motor cortices. (k) Decoding accuracy for the first odour (left two) or choice (right) using N% of selective neurons during the first-odour (left), late-delay (middle), or choice (right) epochs. The analysis is performed with a non-linear LSTM decoder using the identical data from n = 5 mice performing at expert level showcased in Fig. 2e. (l) Maximum decoding accuracy during the first-odour, late-delay, or choice epochs using N% of each epoch selective neurons. Dashed lines represent decoding from shuffled data. (m) Trial category prediction accuracy (AC, AD, BC, BD; n = 5 mice). Chance level, represented by the red trace, is 25% due to the four possible categories. (a)–(m), Mean ± s.e.m. Source Data

As single-neuron activity patterns showed mixed selectivity, we examined whether population decoding techniques could better reveal WM information content represented within the ensemble. We trained a linear support vector machine (SVM) to decode the identity of the first odour from the deconvolved calcium signals of populations of M2 neurons recorded when the animal performed at expert level. When trained with data from all simultaneously recorded neurons, the decoder could successfully decode the odour identity during the odour period and the delay period. SVM decoders could also decode the choice of the animals with high accuracy. To determine whether a small number of selective neurons is sufficient to encode the WM, we investigated how the decoding accuracy changes with the number of neurons used to train the network. We first used the data from the last day of calcium imaging during which all five mice were performing the task at expert level and ranked the selectivity of each recorded neuron. We then trained the decoder using the top *N*% of the most selective neurons, where *N* ∈ {1, 2, 4, …, 64, 100}. We found that the accuracy of decoding the first-odour identity during the first-odour and late-delay epochs and choice during the choice epoch increased with *N* at different rates (Fig. 2e,f). The first-odour identity representation during the late-delay epoch required a much higher number of neurons for adequate decoding than the first-odour identity representation during odour presentation, or the animal’s choice. In further support of this, we performed *t*-distributed stochastic neighbour embedding (*t*-SNE) analysis of the mean activity of neurons to reduce the dimensionality of the population activity during the first-odour, late-delay and choice epochs. The first two *t*-SNE dimensions were sufficient to disentangle the trial type (whether odour A or B was presented during the first odour presentation period) and choice during the first-odour and choice epochs. However, the model could not cluster the trial types from the activity of the late-delay epoch (Extended Data Fig. 3a), reaffirming the notion that late-delay representations were high dimensional. To confirm that the unsuccessful clustering of trial types during the late-delay epoch did not result from the absence of WM representation in the neuronal population activity, we applied SVM decoding to the identical dataset. As illustrated in Extended Data Fig. 3a, SVM decoding successfully identified trial types during the late-delay epoch based on neuronal population activity.

To eliminate the possibility that neuronal activity merely mirrored mouse behaviour, we focused on a subset of neurons exhibiting selectivity during the choice epoch—a crucial period for decision-making in the task. Using these choice-epoch-selective neurons, we attempted to decode the identity of the first odour presented in the task. As shown in Extended Data Fig. 3h, during the late-delay epoch, decoding the first odour identity was not reliably achieved with up to 64% of the choice-epoch selective neurons. However, using all choice-epoch selective neurons (100%) significantly improved decoding performance to 69 ± 3%. This observation suggests that mixed selectivity probably contributes to the decoding process during this epoch.

To determine the specificity of these results, we recorded the activity of M1 and M2, or RSA and M2 neurons simultaneously. We chose the RSA because this region is involved in cognitive tasks^19^ and makes monosynaptic connections with the M2 (ref. ^20^). In expert animals, we could decode the identity of the first odour during the delay period from the activity of the M2 but not the M1 or RSA neurons; the addition of M1 or RSA neurons to M2 neurons did not improve the performance of the decoder beyond what could be decoded from the M2 alone. Even so, we could decode choice from the activity of all three regions (Fig. 2g,h). Thus, successful decoding of odour identity during the late-delay epoch was only possible in the M2.

To assess whether the linearity of the SVM decoder posed limitations on decoding WM during the delay period, we used a nonlinear long short-term memory (LSTM) recurrent neural network decoder for analogous analyses. The performance of the LSTM decoder was very similar to the SVM and did not enhance the decodability of WM information during the delay period or choice during the reward period (Extended Data Fig. 3i–l).

As another control, we conducted calcium imaging recordings of M2 neurons in mice engaged in a go/no-go task that lacked a WM component (Fig. 1h). The mice rapidly acquired proficiency in the task, achieving high performance within a single session. We successfully decoded the identity of the presented odour with a high degree of accuracy (Extended Data Fig. 3b,c). In this task, because the animal has to lick in response to one odour and withhold licking in response to the other, the odour decoding is similar to choice decoding in the delayed association WM task, in which choice decoding exhibited a high level of accuracy for a similar duration of 3 to 4 s subsequent to the introduction of the second odour.

To investigate late-delay activity when the initial odour lacks relevance to the reward, we also conducted calcium imaging of M2 neurons during a WM task with randomized reward contingencies. In this task variant, the reward outcome was not tied to specific odour sequences, and the mice could not discern precise odour combinations associated with the reward. In response to this uncertainty, the mice adopted a strategy of engaging in licking behaviour for all trials, anticipating a water reward in approximately half of them. During the odour presentation and early delay epochs, we could accurately decode the first odour’s identity from the first-odour epoch selective neurons. However, the decoding accuracy progressively declined during the late-delay epoch and beyond, reaching chance levels (Extended Data Fig. 3d). We could not decode the identity of the first odour during the first-odour or late-delay epochs when we used the activity of the late-delay-epoch selective neurons. This decline in decoding accuracy for the first odour can be attributed to the absence of WM representation during the late delay when reward outcomes lack a structured relationship with presented stimuli. The absence of a reward structure prompted consistent licking behaviour in all trials and, notably, we could decode whether the mouse received water (Extended Data Fig. 3d). The decoding accuracy diverged only after a delay following the second odour, presumably once the animal internally processed reward consumption.

To further examine the importance of the late-delay period activity, we compared the accuracy of decoding the identity of the first odour during the late-delay epoch using the activity of the neurons in correct and error trials. The decoding accuracy of the error trials during the first-odour epoch was statistically indistinguishable from the correct trials. However, the decoding accuracy during the late-delay period of the error trials was significantly lower and at chance (54.7 ± 5.2%, *n* = 5) compared with the decoding accuracy of the correct trials (78.5 ± 4.5%; Fig. 2i,j). Thus, population representations of the first odour during the late-delay period are behaviourally relevant.

## Late-delay selectivity emerges after learning

**Fig. 3:**
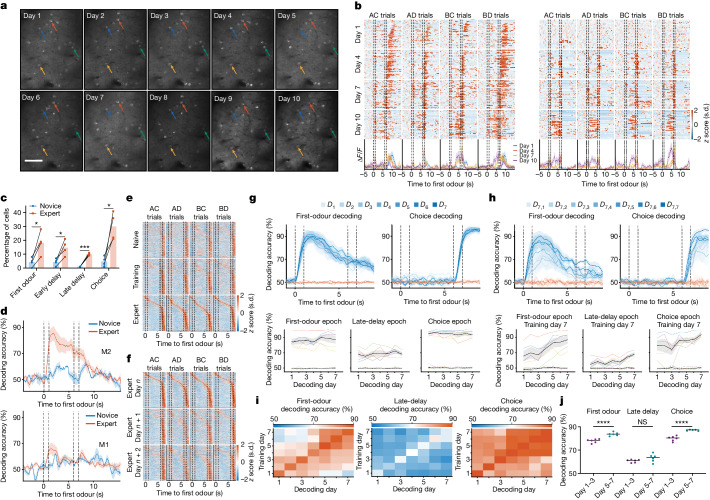
Volatile late-delay epoch WM representation. **a**, Example of the same field of view over 10 days from one of the animals. Images of the other mice looked similar. Scale bar, 100 μm. **b**, *z*-scored activity of two example neurons over 10 days. **c**, The percentage of neurons (*n* = 4 mice) exhibiting selective activity for the first odour during different task epochs (first odour, *P* = 0.023; early delay, *P* = 0.022; late delay, *P* = 0.0002; choice, *P* = 0.026). Statistical analysis was performed using paired *t*-tests. **d**, Improvement in first-odour decoding from M2 and M1 cortices as mice progress from novice to expert stages. *n* = 4 mice. Decoding was performed using overlapping neurons on both novice and expert sessions. All recorded neurons were used for decoding. Novice, the animal’s performance is less than 65%; expert, the animal’s performance is 80% or greater. **e**, The *z*-scored activity of 290 neurons (*n* = 4 mice) across naive, training and expert days, ordered by the response magnitude of the expert day. During the first-odour, early-delay, late-delay and choice epochs, 8.2 ± 5.2%, 6.9 ± 4.5%, 0.2 ± 0.2% and 1.0 ± 0.7% of neurons maintained their selectivity throughout all three stages, respectively. **f**, The *z*-scored activity of 289 neurons (*n* = 4 mice) across 3 days of expert performance, ordered according to the response magnitude of the first day. During the first-odour, early-delay, late-delay and choice epochs, 3.9 ± 2.2%, 4.8 ± 1.7%, 1.0 ± 0.7% and 10.3 ± 4.9% of cells maintained their selectivity across all 3 days, respectively. **g**, Decoding first-odour and choice across 7 days using a daily-trained decoder (top) (*n* = 4 expert mice). Bottom, quantification of decoding accuracy by task epoch; the dashed red lines represent shuffled data accuracy. **h**, Similar to **g**, but the network, trained on day 7 (first subscript value), was tested across all days (1–7; second subscript value). **i**, The decoding accuracy for first odour and choice, trained and tested on all day pairs. **j**, The average decoding accuracies for early versus late days (*n* = 4 mice), showing significant differences (first-odour and choice, *P* < 0.0001; late delay, *P* = 0.12). Statistical analysis was performed using unpaired *t*-tests. For **d**, **g** and **h**, data are mean ± s.e.m. Details of the statistical analyses are provided in the Methods. Source Data

We followed the deconvolved calcium activity of the same population of L2/3 M2 neurons over 10 days and examined the stability of WM neural correlates throughout learning and expert behaviour (Fig. 3a,b). From the first day of training, we observed neurons with significant activity fields during different task epochs (Methods and Extended Data Fig. 2a). A neuron was considered to have significant activity if the distribution of its activity within a specific epoch was significantly different from the distribution of its circularly shuffled activity. In novice mice, 43.3 ± 3.1%, 42.3 ± 3.9%, 52.0 ± 5.5% and 41.3 ± 3.2% of neurons had significant activity fields during the first-odour, early-delay, late-delay and choice epochs, respectively (*n* = 4 mice). As the mice learned the task, more neurons were recruited during all time epochs. In expert mice, the proportion of neurons with significant activity fields during first-odour, early-delay, late-delay and choice epochs increased to 58.5 ± 3.7%, 68.5 ± 2.5%, 61.3 ± 6.0% and 54.3 ± 2.5%, respectively (Extended Data Fig. 2a).

As mice learned the task, the proportion of neurons that fired selectively to odour A or B during the first-odour, early-delay and late-delay epochs, and lick or no-lick during choice epoch significantly increased (Fig. 3b,c and Extended Data Fig. 2e). Importantly, odour-selective firing during the late-delay epoch emerged only after the mice learned the task.

In the RSA and M1, the proportion of selective neurons during the first-odour, early-delay and late-delay epochs did not increase with learning. However, the proportion of cells activated during the choice epoch increased in the expert phase in both the M1 and RSA (Extended Data Fig. 2j,k).

To determine whether individual neurons encode the association of rewarded odours, we examined whether any neurons were selectively activated by associated pairs of rewarded odours (odours A and C, or odours B and D). In novice mice, no cells demonstrated selectivity for both odours A and C, or both odours B and D. By contrast, among expert mice, only 1.4 ± 0.5% exhibited selectivity for both odours A and C, or both odours B and D. Consistent with the presence of these neurons, we were able to train a decoder to discriminate between trial categories (AC, AD, BC or BD) during the second odour using population activity patterns (Extended Data Fig. 3m).

## Late-delay WM representation emerges after learning

To determine how WM population representations emerge with learning, we used the activity of the same overlapping population of neurons when the mouse was a novice and after it became an expert in the task to train the network and decode for each day separately. In novice mice, the cross-validated first-odour identity decoding accuracy during the first-odour and late-delay epochs were at chance level. Conversely, in expert animals, the accuracy of first-odour identity decoding during the first-odour and late-delay epochs significantly increased to 80.3 ± 5.3% and 73.0 ± 4.2%, respectively (Fig. 3d). To determine whether WM-related activity was specific to the M2 or was present within other structures implicated in motor execution or rule-based task performance^21^, we performed a similar analysis in recorded neurons in the M1. The decoder performed significantly worse, with a decoding accuracy of near chance (53.6 ± 2.4%) in novice and 64.7 ± 4.0% in expert mice during the first-odour epoch. During the late-delay epoch, the accuracy of decoding the first odour identity from M1 neurons was near chance levels at both the novice and expert stages of task performance (Fig. 3d).

## Stabilization of activity patterns over days

Our analyses demonstrated gradual and pronounced changes in M2 neuronal responses across days (Fig. 3b). We quantified the specificity of neurons for different parameters during different task epochs at different learning stages (Extended Data Fig. 2e,f). Single neurons either gained or lost responsiveness to a specific odorant and rarely exhibited stable responses across sessions. First-odour information during the late-delay epoch emerged as the animals became experts in task performance. Critically, changes in the responses of individual neurons were due to continual alterations in responses across sessions, not to a global loss of responsiveness in the M2.

To understand how stable the WM representation was across days, even after the animal became an expert, we tracked the activity of 290 cells (*n* = 4 mice) during the learning phase in naive, training and expert mice (Methods). In total, 8.2 ± 5.2% of tracked neurons maintained their selectivity during the first-odour epoch throughout all three stages (Fig. 3e). For the early-delay, late-delay and choice epochs, 6.9 ± 4.5%, 0.2 ± 0.2% and 1.0 ± 0.7% neurons remained stable. During the expert phase (Fig. 3f), we tracked the activity of 289 cells (*n* = 4 mice) across three consecutive days; we found that only 3.9 ± 2.2%, 4.8 ± 1.7%, 1.0 ± 0.7% and 10.3 ± 4.9% of cells maintained their selectivity during first-odour, early-delay, late-delay and choice epochs across all 3 days, respectively. Thus, neurons with significant late-delay activity were the least stable during the expert phase.

To determine whether population representations of L2/3 M2 activity were stable during the expert stage, we used same-day and cross-day decoding approaches. At the population level, we determined whether decoders trained and tested on the same-day activity of expert animals could reliably predict the first odour and choice for seven consecutive days using activity recorded from L2/3 M2 neurons. The decoding accuracy for all epochs was high and remained stable across all 7 days (Fig. 3g). There were no significant changes in performance, recorded neuron count or the percentage of neurons exhibiting selectivity across imaging days for the analysed mice (Extended Data Fig. 3e–g). To quantify the stability of network dynamics across days, we tested whether the network that was trained to decode the identity of the first odour or choice on the expert day seven could also decode the first-odour identity or choice on the earlier days. For this analysis, we used the activity of overlapping populations of neurons on both days. If the same population of neurons shared a common representation and dynamics across days, we would expect the decoding accuracy to be independent of the day the decoder was tested. The accuracy of decoding the first odour during the first-odour epoch gradually declined over days but remained above chance (Fig. 3h). The accuracy of choice decoding declined slowly, like that of odour decoding during the first-odour epoch. By contrast, late-delay representations drifted more rapidly to chance. To determine whether stability emerged gradually during the expert performance period, we conducted a similar analysis but trained on each of the 7 days and compared the performance on the remaining 6 days (Fig. 3i). Our null hypothesis assumed that the decoding stability remains the same across all 7 days. To assess this, we used the cross-day decoding accuracy statistic averaged across animals for late versus early days. The decoding accuracy of the identity of the first odour during the first-odour epoch and choice during the choice epoch when decoders were trained and tested on days 5–7 was significantly higher compared with when the training and testing were performed on days 1–3, rejecting the null hypothesis (Fig. 3j). No such pattern was evident for late-delay epoch decoding of the first odour. As a secondary measure, we performed an additional statistical test by shuffling the day identities. If the decoders show no greater stability at later days compared to earlier ones, our statistics should be similar to the one obtained by shuffling day identities. However, we observed that this is not true for the first-odour and choice epochs. Consequently, the results of this second test also rejected the null hypothesis that decoding stability remains constant across all 7 days (Extended Data Fig. 2n). To determine whether this trend was stable across animals, we compared the mean decoding accuracy within individual mice across first and last 2 day pairs (Extended Data Fig. 2l). Decoding showed a statistically significant improvement during the choice epoch. Thus, as the mice continue to perform the task (even as experts), their representations for the first odour and choice epochs stabilize, while representations for the late delay seem to drift daily.

## Emergence and crystallization of late-delay epoch WM representation

As shown above, the activity of L2/3 neurons in expert animals demonstrated considerable representational drift during the late-delay epoch. We therefore examined whether this drift was layer specific, and whether deeper layers hold an invariant memory of representation across days. To answer this question, we performed volumetric imaging of tens of thousands of M2 neurons during the expert performance to investigate whether the late-delay representation stabilizes with continued practice.

**Fig. 4:**
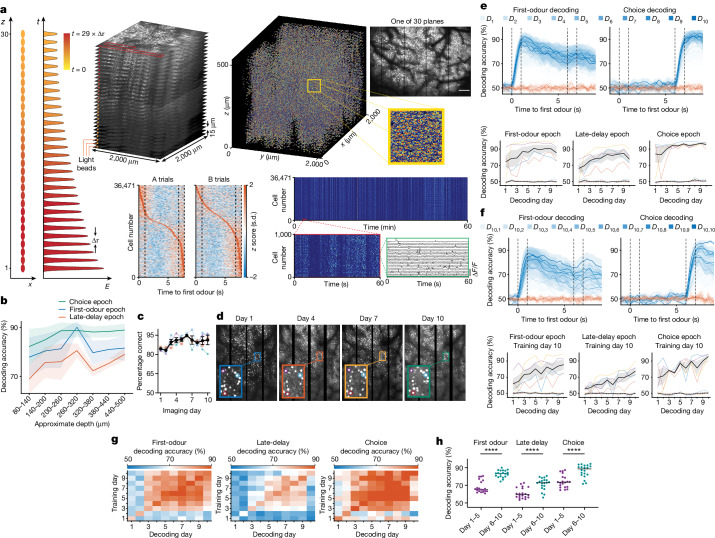
The stability of late-delay epoch representation. **a**, Schematic of LBM (left). A pump pulse is split into 30 light beads, each delayed (Δ*τ *≈ 7 ns, where Δ*τ* represents the time delay between excitation foci) and focused at different sample depths, enabling full-volume sampling at the microscope’s frame rate. Top right, a 3D rendering of neuron locations across 30 planes. Bottom middle, the activity of 36,471 neurons during A-odour or B-odour trials, sorted by response and shown over 60 min. Bottom right, a 60 min raster of these neurons, highlighting Δ*F*/*F* activity for selected neurons. Scale bar, 500 μm. **b**, The accuracy of decoding the first odour and choice during the first-odour, late-delay and choice epochs using the activity of neurons at different depths. *n* = 4 mice. **c**, The performance of the mouse during the 10 days of calcium imaging. Mice (*n* = 4) were performing at expert level from the first day of imaging. **d**, Example field of view showing 1 out of the 30 planes with the same constellation of neurons across multiple imaging days. **e**, Decoding first odour and choice over 10 days with a daily-trained decoder, using up to 73,307 neurons across 30 planes and 450 μm depth (top) (*n* = 4 mice). Bottom, quantification of accuracy by epoch; the dashed lines indicate shuffled data decoding. **f**, Similar to **e**, with the decoder trained on day 10 and tested across days 1–10. **g**, The decoding accuracy for first odour during first-odour and late-delay epochs, and choice accuracy during the choice epoch. *n* = 4 mice. **h**, The average across-day decoding accuracies for early versus late days (*n* = 4 mice), as depicted in the off-diagonal terms of Fig. 4g, with significant improvements (first-odour and late-delay, *P* < 0.0001; choice, *P* = 0.0003). Statistical analysis was performed using unpaired *t*-tests. For **b**, **c**, **e** and **f**, data are mean ± s.e.m. Details of the statistical analyses are provided in the Methods. Source Data

We recorded the activity of up to 73,307 M2 neurons simultaneously in a volume of 2,000 × 2,000 × 450 μm^3^ using light-bead microscopy (LBM)^22^, while TRE-GCaMP6s^23^ mice performed the task (Fig. 4a). LBM uses two-photon excitation of axially separated and temporally delayed excitation foci to record from 30 axially separated voxels over 500 μm within 200 ns. Lateral scanning and temporal demultiplexing of the signal from this column of light beads results in 6.5 Hz recording of neuronal activity within 30 imaging planes, each separated by 15 μm, extending from superficial L2/3 to superficial L5. We examined the dependence of decoding accuracy as a function of recording depth. We divided the bottom 420 μm depth of our calcium imaging volume into seven intervals of 60 μm thickness each. We identified the recorded neurons in all seven intervals and noted the layer with the fewest number of neurons recorded. We equalized the number of neurons analysed for decoding by randomly selecting neurons to match the number of neurons recorded in this layer containing the fewest neurons. We then calculated the accuracy of decoding the identity of the first odour or choice during the first-odour, late-delay or choice epochs. The accuracy of the first-odour decoding during the first-odour epoch initially increased with increasing depth and then decreased to an intermediate level (Fig. 4b). However, the accuracy of decoding the first odour during the late-delay epoch increased monotonically with increasing depth. The accuracy of decoding choice during the choice epoch was uniformly high at all depths. There was therefore an increase in information about the first odour during the late-delay period in the deeper cortical neurons imaged.

To determine whether individual neurons encode the association of rewarded odours, we examined whether any neurons were selectively activated by associated pairs of rewarded odours (odours A and C, or odours B and D). In expert mice, only 1.2 ± 0.5% of neurons exhibited selectivity for both odours A and C, or both odours B and D. The sequences of these cell activities during AC and BD trials, sorted on the basis of their activity in both trial types and vice versa, revealed the presence of numerous cells with mixed selectivity for A and B odours or C and D odours. Importantly, the sequences differed between the two trial types, underscoring that neurons convey content-specific information during the delay period. While some neurons exhibited a strong correlation in activity during the AC and BD trials, most did not show a significant correlation. Particularly, the correlation was weakest at the point at which the delay period sequence was formed, indicating greater dissimilarity in activity during the delay period across different trial types (Extended Data Fig. 2p).

**Extended Data Fig. 4:**
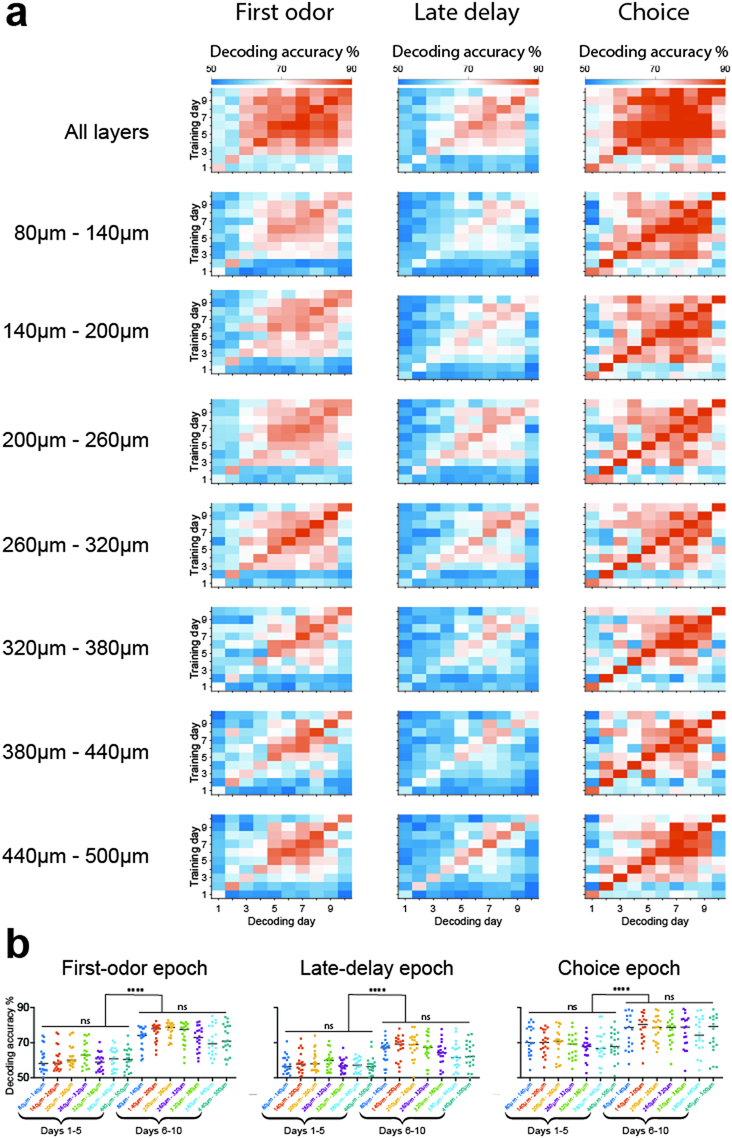
Cortical depth-dependent neural decoding accuracy of the first-odour identity and choice in mice across task epochs. (a) Accuracy of decoding the first odour identity during the first-odour (left column) and late-delay (middle column) epoch using neurons at different depths. The right column is the accuracy of decoding choice during the choice epoch. (b) Mean decoding (n = 4) accuracy obtained by training the decoder on data from a specific depth on one of the five days (1–5 or 6–10), followed by testing the decoder on the data of the same depth from the remaining four days. Each data point represents the outcome of one of the 20 permutations (one-way ANOVA; first-odour epoch, *p* < 0.0001; late-delay epoch, *p* < 0.0001; choice epoch, *p* < 0.0001). *p≤ 0.05, **p≤ 0.01, ***p≤ 0.001, ****p≤ 0.0001, ns, not significant. See Methods for details of statistical analyses. Source Data

We wondered whether continued practice refines the WM representation of the first odour, pushing the neuronal activity to a single attractor, stable across days. To investigate this, we followed the deconvolved calcium activity of the same population of neurons over 10 days of expert performance (Fig. 4c,d) and examined the stability of WM neural correlates throughout expert behaviour. We took great care to match the identity of neurons recorded with volumetric imaging across days, matching up to 47,384 neurons across days. Consistent with our earlier results, when the decoder was trained and tested on the activity from the same day of recording, the identity of the first odour could be decoded with high accuracy during the first-odour and late-delay periods, and choice could be decoded with very high accuracy during the choice epoch (Fig. 4e). Also consistent with our earlier results, the identity of the first odour and choice could be decoded with relatively high accuracy when the decoder was trained on the activity of day 10 (the final day of imaging) and tested on previous days of imaging, with the decoding accuracy declining with an increasing number of days between training and testing days; this indicated the emergence of a stable representation during the final imaging days (Fig. 4f,g). However, in contrast to our previous results using single plane L2/3 imaging, the performance of decoders in predicting the first odour during the late-delay epoch was higher when trained and tested on days 6–10 of imaging compared with when trained and tested on days 1–5 of imaging (Fig. 4g,h). We conducted an additional statistical test by shuffling the day identities. If decoding stability is not higher in the later days compared to earlier ones, our results should resemble those obtained by randomly shuffling day identities. However, we observed that this is not the case for any of the three epochs. Consequently, our second test also rejected the null hypothesis, indicating that decoding stability does not remain constant across all 10 days (Extended Data Fig. 2m). Moreover, to examine whether this trend holds across different animals, we compared the mean decoding accuracy within individual animals for the first and last 3 days pairs (Extended Data Fig. 2o). The decoding accuracy demonstrated a statistically significant improvement during all three epochs. In other words, during the first 5 days of expert performance, the decoder trained on the activity on any day’s imaging could not decode the identity of the first odour during the late-delay epoch on any other day. However, as the mouse kept practicing the task, during days 6–10 of expert performance, the decoder trained on the activity of neurons during any of these later days could accurately decode the identity of the first odour during the late-delay epoch. Thus, in addition to the stabilization of activity during the first-odour and choice epochs, volumetric imaging demonstrated stabilization of the WM representation with continued practice during the late-delay epoch. The stabilization was uniform throughout the imaged volume (Extended Data Fig. 4).

## Discussion

Tracking M2 neuron activity over 10 days revealed that late-delay WM representations, which are crucial for task performance, initially fluctuate in early expert stages but stabilize with continued practice in the late expert phase.

M2 neuron activity strongly encoded odour identity in the early task stages without affecting behaviour when disrupted. This suggests that early-stage activity does not drive persistent attractors within the M2. The sensitivity to disruption emerges in late-delay and choice epochs, indicating that late-delay activity probably depends on interactions with regions projecting to the M2. This allows for recovery and realignment to the attractor state by the late-delay period despite early disruptions.

Inhibiting M2 neurons during late delay in training animals did not impair rule learning, suggesting that task learning occurs upstream of the secondary motor cortex. Alternatively, neural redundancy might explain this, whereby inhibiting one area could be compensated by other regions or pathways that are involved in the task.

Our study extends previous research performed in the premotor cortex during motor preparation and WM tasks. It was previously shown that activity in the premotor anterior lateral motor cortex during the first-odour and delay periods was important for task performance^24^. Differences in findings between our study and theirs may arise owing to differences in delay duration (1.5 s versus 5 s for our study) and differences in recording location (anterior lateral motor cortex versus M2 in our study). Chronic recordings from the same population of neurons over days to weeks have enabled investigators to track whether a population of neurons in cortical or hippocampal regions encodes sensory, motor or cognitive variables in a stable manner or whether these distributions drift over time^25–34^. In general, while there was variability in the engagement or selectivity of individual neuron firing rates from day to day (but see refs. ^32–35^ for highly stable reactivation), in most cases, reliable cross-day population decoding of sensory and cognitive variables was still possible for several days up to several weeks^25,28,30,36^. In contrast to these findings, for late-delay decoding, we saw the emergence of successful cross-day decoding only after several days of performance of the task in mice already performing the task at high accuracy levels. This reassignment of neurons participating in late-delay activity from day to day during learning and early expert performance periods may allow more rapid erasure of the WM sketchpad and may be necessary for increasing WM capacity for the large number of stimuli encountered in the world. It may also allow more flexibility for adapting to new decision-making rules as WM representations are recruited for driving distinct actions in different contexts. Future studies will dissect plasticity processes that drive dynamic learning-related activity patterns that stabilize with continued practice.

## Methods

### Mice

All of the experiments were conducted according to the National Institute of Health (NIH) guidelines and with the approval of the Chancellor’s Animal Research Committee of the University of California, Los Angeles. Experiments were performed with 8–15-week-old adult male and female C57BL/6J (Jackson Laboratory, 000664), C57BL/6J-Tg (Thy1-GCaMP6s), GP4.12Dkim/J (Jackson Laboratory, 025776) and B6;DBA-Tg(tetO-GCaMP6s)2Niell/J (Jackson Laboratory, 024742) mice crossed with B6.Cg-Tg(Camk2a-tTA)1Mmay/DboJ (Jackson Laboratory, 007004) mice. Mice were kept in the vivarium under a 12 h–12 h light–dark cycle.

### Viruses

For optogenetic experiments, CaMKIIa-driven soma-targeted anion-conducting channelrhodopsin fused to FusionRed (pAAV-CKIIa-stGtACR2-FusionRed, Addgene, 105669; titre, 1 × 10^13^ viral genomes per ml) was used to express GtACR2 in the soma of excitatory neurons. For control experiments, we used pAAV-CaMKIIa-mCherry (Addgene, 114469; titre, 7 × 10^12^ viral genomes per ml) or pAAV-CaMKIIa-EGFP (Addgene 50469; titre, 7 × 10^12^ viral genomes per ml).

### Head-bar and cranial window implantation

Adult 8-to-12-week-old male and female C57BL/6J-Tg (Thy1-GCaMP6s) GP4.12Dkim/J mice were anaesthetized with isoflurane (5% for induction, 1–2% (v/v) for maintenance). The depth of anaesthesia was monitored continuously and adjusted when necessary. After induction of anaesthesia, the mice were fitted into a stereotaxic frame (Kopf), with their heads secured by blunt ear bars and their noses placed into an anaesthesia and ventilation system (David Kopf Instruments). Body temperature was kept at 37 °C with a feedback-controlled heating pad (Harvard Apparatus). Mice were administered 0.05 ml lidocaine (2%; Akorn) subcutaneously as a local anaesthetic before surgery. The surgical incision site was cleaned three times with 10% povidone-iodine and 70% ethanol. After removing the scalp and clearing the skull of connective tissues, a custom-made lightweight metal head-bar was fixed onto the skull with cyanoacrylate adhesive and covered with black dental cement (Ortho-Jet). A circular craniotomy (diameter, 5 mm) was performed above the secondary motor cortex (centred at 1.94 mm anterior from bregma or centred at bregma for M2/RSA imaging). A cranial glass window consisting of a 5 mm diameter round #1 coverslip (Warner Instruments) was implanted in the craniotomy, flush with the skull surface and sealed in place using tissue adhesive (Vetbond). The exposed skull surrounding the cranial window was then completely covered with black dental cement to build a small chamber for imaging with a water-immersion objective. After surgery, the mice were injected with carprofen (5 mg per kg of body weight) and allowed to recover overnight in cages placed on a low-voltage heating pad. Carprofen was administered once per day for up to 2 days after surgery. Amoxicillin antibiotic (0.25 mg ml^−1^) was dispensed in the drinking water for 7 days. Animals were returned to the vivarium for 1–2 weeks for recovery before undergoing imaging experiments.

### AAV injection and fibre optic cannula implantation

Adult 8-to-12-week-old male and female C57BL/6J mice were anaesthetized with isoflurane (5% for induction, 1–2% (v/v) for maintenance). Skin incisions were made, followed by craniotomies 1 mm in diameter above the secondary motor cortex (centred at 1.94 mm anterior to bregma and 0.5 mm lateral to the midline) using a small steel burr (Fine Science Tools) powered by a high-speed drill. Saline (0.9%) was applied to the skull to reduce heating caused by drilling. Bilateral viral injections were performed by using stereotaxic apparatus (David Kopf Instruments) to guide the placement of bevelled glass pipettes with a tip diameter of about 50 μm (World Precision Instruments) into the secondary motor cortex (1.94 mm anterior to bregma, 0.5 mm lateral to the midline and 0.3 mm from the pial surface). Using the Nanoject II micro-injector (Drummond Scientific), 300 nl of 1:100 PBS-diluted AAV was bilaterally injected using a syringe pump. Glass pipettes were left in place for at least 10 min after virus injection.

A ferrule-terminated optical fibre (Thorlabs) was placed above the injected site. The fibre tip was aimed to terminate at the pial surface. The optical fibre was secured to the skull using cyanoacrylate adhesive and black dental cement (Ortho-Jet). After surgery, the mice were left overnight in cages placed on a low-voltage heating pad. Mice were allowed to recover for 2–3 weeks before the experiments. The locations of injections and implanted optical fibres were validated histologically for all experimental mice.

### Behavioural training

After recovery from surgery, mice were handled and water-restricted to 85–90% of their original weight. The mice were subsequently habituated to head fixation, airflow and water port for two sessions (one session per day). During the two shaping days, the mice were presented only with the combination of the odours (A, 1-pentanol; B, butyl formate; C, 3-methyl-2-buten-1-ol; and D, ethyl acetate; Sigma Aldrich, 138975, 261521, 162353 and 270989) that led to reward (AC and BD trials) and water was automatically delivered. After 2 days of shaping, the mice were trained to perform the complete delayed-association WM task. The lick port was connected to a touch sensor, and mouse tongues had to touch the lick port at least once to receive a water reward. Each training session consisted of 150 to 250 trials. Odour combinations were presented in a random order. Responses were assessed based on mouse licking during the choice window. If any licks occurred during the choice window, the trial was considered to be a hit for AC and BD trials or false alarm for AD and BC trials. If no licking occurred during the choice window, the trial was considered to be a miss for AC and BD trials or correct rejection for AD and BC trials. Mice were not punished for miss or false alarm trials. A training session was aborted early if a mouse had more than three misses within the most recent ten trials, indicating the animal’s lack of motivation to obtain the water reward. Performance was quantified as the number of hits and correct rejections over the total number of completed trials. The airflow and odour delivery were frequently monitored using an Aurora Scientific photo-ionization detector at the beginning of each training session.

### In vivo calcium imaging

Two-photon laser-scanning microscopy was conducted using the Thorlabs multiphoton mesoscope using a 12 kHz resonant scanner with a water-immersion objective with 0.6 excitation NA, 1.0 collection NA and 2.7 mm working distance. The excitation laser was a 920 nm Tiberius Ti:Sapphire Femtosecond Laser, and the laser intensity was 30–80 mW at the sample. Images were acquired using the ScanImage software (Vidrio Technologies). Fully awake mice were mounted in a 2-inch-diameter transparent tube by securing its head bar onto a custom-made head-bar holder under the microscope. 600 px × 1,200 px to 600 px × 2,500 px images were acquired at 8–17 Hz at 150–250 μm depth. To track the mouse movement, a camera mounted underneath the animal acquired the paw location of the animals at 30 Hz. The locomotion data were acquired simultaneously with the calcium imaging data and synchronized with the scanning mirror signals. The microscope and behavioural set-up were encased in a light-tight box, and the mice were kept in darkness during the imaging sessions. We performed online image processing at the beginning of every session to align cells across days. We tried to maximize the correlation between the moving average of frames of the current field of view and the average of frames of the previous sessions.

Two-photon LBM was conducted using a custom-built microscope equipped with a 960 nm, 4.89  MHz repetition rate optical parametric chirped-pulse amplification (OPCPA) pumped by an ytterbium laser at 1,030 nm with 80 W power, delivering a 2 μJ pulse energy and a 90 fs pulse width. The LBM featured a rapid 12 kHz resonant scanner and was paired with a 0.6 excitation NA, 1.0 emission water-immersion objective lens with a 2.7 mm working distance. The LBM technique divided a single pulse into 30 distinct subpulses of varying intensities, targeting 30 separate depths of the specimen separated by 15 μm, yet eliciting a consistent level of fluorescence across these layers^22^. In our initial LBM experiments, we successfully recorded a region measuring 1,450 × 1,825 × 450 μm^3^ at a frequency of 7.95 Hz in two mice. Subsequent experiments extended the recorded area to 2,000 × 2,000 × 450 μm^3^, recorded at 6.45 Hz, in another two animals.

### Optogenetics

Optical stimulation was applied through a ferrule-terminated 200 μm core and 0.39 NA optical fibre (Thorlabs) attached to the 200 μm core and 0.39 NA patch cable using a 1.25 mm ceramic mating sleeve (Thorlabs). We used a blue-fibre-coupled light emission diode (*λ* = 470 nm, Thorlabs, M470F3). The light was delivered at 20 Hz with a 0.4 duty cycle at an irradiance of 10 mW mm^−2^ at the output tip of the fibre.

Optogenetic experiments commenced only when the animals achieved a behavioural performance threshold exceeding 90% accuracy for at least three consecutive sessions. This criterion ensured that the animals were well-trained and proficient in reliably executing the behavioural tasks before the introduction of optogenetic manipulations.

### Electrophysiology

For in-vivo electrophysiology recordings, expert mice were anaesthetized with isoflurane (5% for induction, 1–2% (v/v) for maintenance). They underwent a 2 mm craniotomy (centred at 1.94 mm anterior to bregma and 0.5 mm lateral to the midline) and silver wire ground (Warner Instruments) implantation surgery over the cerebellum 1 day before recording. The ground wire was fixed in place with dental cement. The exposed skull was covered with Kwik-Sil, and the mouse was allowed to recover overnight. On the day of the recording, the mice were head-fixed into a tube, the Kwik-Sil covering the craniotomy was removed and replaced with buffered artificial cerebrospinal fluid, and the mouse was aligned to the micromanipulator. A 128-channel silicon microprobe^37^ was slowly lowered using a micromanipulator into M2, and the surface of the exposed brain was covered with mineral oil. The process was monitored using a surgical microscope (Zeiss, STEMI 2000). The microprobe contained 128 channels that were densely distributed (honeycomb layout with 20 μm spacing between nearest-neighbour channels) on two shanks (placed 0.4 mm apart). After insertion, the microprobe was allowed to settle for at least 30 min before the recording began and continued for the entire duration of the session. The electrophysiological and behavioural data acquisitions were synchronously performed using custom MATLAB software while the mouse performed the task. The probe readout was achieved using a detachable head stage module (Intan Technologies RHD 128). Head stages contained commercial integrated electronic circuits (Intan Technologies RHD 2000 USB interface board) providing a multiplexed signal recorded with open source software (Intan Technologies) at 25 kHz per channel.

### Histology

At the end of experiments, the mice were deeply anaesthetized under isoflurane and transcardially perfused with 40 ml 1× PBS followed by 40 ml 4% paraformaldehyde in 1× PBS at a rate of approximately 4 ml min^−1^. After perfusion, the brains were rapidly extracted and post-fixed in 4% paraformaldehyde. Coronal sections (thickness, 100 μm) were collected using a vibratome. The sections were mounted onto glass slides. The slides were then cover-slipped with mounting medium DAPI. Images were acquired using the Leica DM6 B microscope.

### Quantification and statistical analysis

Calcium imaging data processing, including motion correction, segmentation, fluorescence signal extraction and deconvolution, was performed using the Python implementation of Suite2P^38^. Before segmentation, we performed several steps to enhance image quality, including noise reduction, background subtraction and image registration to correct for tissue movement. We validated our segmentation results by comparing the automated segmentation to manually annotated ground truth data. Adjustments to parameters and algorithms were done to achieve optimal results. We used the deconvolved signal for all our analyses. Silicon probe data processing and spike sorting were performed using custom code, KiloSort^39^ and Phy^40^.

To visualize the calcium activity of individual neurons, we computed a peristimulus time histogram averaged across all trials for all four combinations of odours, smoothed using a moving average over a 400 ms window. To generate response maps for each neuron, we subtracted its mean spontaneous baseline calcium activity across all trials on a given day during the baseline epoch (5 s before the first-odour onset). We divided it by the s.d. of calcium activity during the baseline epoch. Thus, the response maps show changes in calcium activity in units of the s.d. of spontaneous activity. This method was used for visualization purposes only. Unless stated otherwise, all statistical analyses were performed on unsmoothed, deconvolved calcium activity without baseline calcium activity subtracted.

A neuron was considered to have a significant activity field during a specific time epoch if its activity within that epoch significantly differed from the distribution of its 1,000 times circularly shuffled mean activity.

The first-odour selectivity of a neuron was assessed by comparing the distribution of its mean deconvolved calcium activity over a time epoch for A and B odour trials using the Wilcoxon rank-sum test with a confidence interval of 99%. A neuron was considered to be purely selective if it exhibited selectivity for a specific odour or choice during a specific epoch and did not show selectivity for any other parameter at any other time. Conversely, a neuron was considered to be mixed selective if it showed selectivity for more than one odour or choice at different epochs.

We considered an animal naive, training or expert if its behavioural performance (*p*) was, respectively, *p* < 65%, 65% ≤ *p* < 80% or *p* ≥ 80%. An animal was considered a novice during the first training day.

To determine whether a neuron’s response was related to the animals’ motor activity, we used DeepLabCut^41^ to find the position of the animals’ paws from which we extracted the animals’ movements. We calculated the correlation coefficient between the activity of each neuron and the unshuffled and 1,000 times circularly shuffled locomotion activity. A neuron was considered to be significantly correlated if its correlation coefficient was at least 2 s.d. away from the mean value of the correlation coefficients of shuffled distribution.

We assessed the WM information content in a population activity of neurons by measuring the classification performance using a SVM with a linear kernel. We implemented the SVM binary classification in MATLAB and performed the computations on high-performance computing clusters using thousands of computing nodes. We used the activity of neurons in 500 ms time bins to train a decoder on 90% of randomly chosen trials and tested its accuracy on the 10% of the trials that were withheld. To ensure our model was not biased or overfit to specific data patterns, we repeated the classification measurements at least 32 times with different sets of randomly chosen trials. We then calculated the average of all measurements. This approach introduces randomness and helps to ensure that the decoding results are not a product of a model memorizing specific instances. Decoding accuracy and its standard error were then found by averaging the prediction accuracy of the decoder across all mice.

For across-day classification, we used the activity of the overlapping neurons for our analyses. We trained a model on all trials on one day and tested that model’s predictions on all trials on another day. To assess statistical significance and determine whether decoding performance surpasses chance, we randomized trial types by assigning random labels to each trial.

For the LSTM decoding analyses, we configured the recurrent neural network architecture so that the dimensions of the input layer were aligned with the number of neurons recorded in the pertinent dataset. The network comprised 128 hidden units followed by a linear layer to compute logits for a softmax classifier using cross-entropy loss. The weights and biases within the LSTM layers during the training phase were optimized using the adaptive moment estimation (Adam) optimizer. LSTMs were trained for 100 epochs (passes through the training set). For the decoding process, temporal data granularity was 500 ms. Training involved 90% of randomly selected trials, with the remaining 10% reserved for testing. Analogous to the SVM decoding procedure, the LSTM classification was iterated at least 32 times using distinct randomly chosen trial subsets. The resultant metrics were then averaged over the 32 samples. Furthermore, we performed trial shuffling to mitigate potential biases and ensure the robustness of the LSTM model’s performance assessment.

To find overlapping neurons across sessions, we used co-registration of spatial cell footprints using CellReg^42^. Neurons were modelled with a maximal centroid distance of 10 μm. Final registration used the probabilistic model with a threshold of more than 95% probability of cells being the same for all mice. We used these parameters to ensure the accuracy of matching cells.

We used *t*-SNE^43^ to embed the high-dimensional neuronal activity into two dimensions. We calculated the time-averaged calcium activity of neurons during a specific epoch and found the pairwise distances between the high-dimensional points for each trial. For each point, we calculated a s.d. so that the perplexity of each data point matched a predefined value. Starting from an initial set of low-dimensional points, we iteratively updated the points to minimize the Kullback–Leibler divergence between a Gaussian distribution in the high-dimensional space and a *t*-distribution in the low-dimensional space.

### Statistics and reproducibility

All statistical analyses were conducted using Prism (GraphPad), MATLAB (MathWorks) or Python. Statistical tests used in this study include Wilcoxon rank-sum tests and paired *t*-tests. The significance threshold was held at *α* = 0.05; NS, not significant (*P* > 0.05); *P ≤ 0.05, **P ≤ 0.01, ***P ≤ 0.001, ****P ≤ 0.0001. All behavioural, imaging and optogenetics experiments were replicated in multiple animals. Sample sizes were not predetermined using statistical methods.

### Reporting summary

Further information on research design is available in the Nature Portfolio Reporting Summary linked to this article.

## Online content

Any methods, additional references, Nature Portfolio reporting summaries, source data, extended data, supplementary information, acknowledgements, peer review information; details of author contributions and competing interests; and statements of data and code availability are available at 10.1038/s41586-024-07425-w.

### Supplementary information

Reporting Summary

### Source data

Source Data Fig. 1

Source Data Fig. 2

Source Data Fig. 3

Source Data Fig. 4

Source Data Extended Data Fig. 1

Source Data Extended Data Fig. 2

Source Data Extended Data Fig. 3

Source Data Extended Data Fig. 4

## Data Availability

All data and analyses necessary to understand the conclusions of the manuscript are provided in the Article. Source data are provided with this paper.

## Code availability

Calcium imaging data analysis (https://github.com/MouseLand/suite2p, https://github.com/flatironinstitute/NoRMCorre and https://github.com/zivlab/CellReg), electrophysiology data analysis (https://github.com/MouseLand/Kilosort and https://github.com/cortex-lab/phy), animal paw tracking (https://github.com/DeepLabCut/DeepLabCut) and decoding analysis (https://github.com/bellafard/DAT-Decoding) are available at GitHub.
