# Supplementary material for: Volatile working memory representations crystallize with practice
Source: Nature. 2024 May 15;629(8014):1109–17. doi: 10.1038/s41586-024-07425-w (PMC11136659; doi:10.1038/s41586-024-07425-w)
Supplement: Supplementary file 1 — Reporting Summary [file 41586_2024_7425_MOESM1_ESM.pdf]

nature portfolio

Corresponding author(s): Arash Bellafard

Last updated by author(s): Mar 18, 2024

Reporting Summary

Nature Portfolio wishes to improve the reproducibility of the work that we publish. This form provides structure for consistency and transparency in reporting. For further information on Nature Portfolio policies, see our Editorial Policies and the Editorial Policy Checklist.

Statistics

For all statistical analyses, confirm that the following items are present in the figure legend, table legend, main text, or Methods section.

n/a	Confirmed	
☐	☒	The exact sample size (*n*) for each experimental group/condition, given as a discrete number and unit of measurement
☐	☒	A statement on whether measurements were taken from distinct samples or whether the same sample was measured repeatedly
☐	☒	The statistical test(s) used AND whether they are one- or two-sided<br>*Only common tests should be described solely by name; describe more complex techniques in the Methods section.*
☐	☒	A description of all covariates tested
☐	☒	A description of any assumptions or corrections, such as tests of normality and adjustment for multiple comparisons
☐	☒	A full description of the statistical parameters including central tendency (e.g. means) or other basic estimates (e.g. regression coefficient) AND variation (e.g. standard deviation) or associated estimates of uncertainty (e.g. confidence intervals)
☐	☒	For null hypothesis testing, the test statistic (e.g. F, t, r) with confidence intervals, effect sizes, degrees of freedom and P value noted<br>*Give P values as exact values whenever suitable.*
☒	☐	For Bayesian analysis, information on the choice of priors and Markov chain Monte Carlo settings
☒	☐	For hierarchical and complex designs, identification of the appropriate level for tests and full reporting of outcomes
☐	☒	Estimates of effect sizes (e.g. Cohen's *d*, Pearson's *r*), indicating how they were calculated

Our web collection on statistics for biologists contains articles on many of the points above.

Software and code

Policy information about availability of computer code

Data collection	Imaging data were partially collected using commercially available two-photon mesoscope from Thorlabs and partially collected using the light bead microscope that is previously published (Demas et. al. 2021, Nature Methods). Behavioral data were collected using National Instruments data acquisition device. Electrophysiological data were collected using commercially available Intan Technologies headstage and circuit board.
Data analysis	Calcium imaging data processing was performed using the Suite2P (https://github.com/MouseLand/suite2p), NoRMCorre (https://github.com/flatironinstitute/NoRMCorre) and CellReg (https://github.com/zivlab/CellReg). Electrophysiology data was performed using KiloSort 2.5 (https://github.com/MouseLand/Kilosort) and Phy2 (https://github.com/cortex-lab/phy). Confocal images were analyzed using ImageJ 1.53q. Analysis codes (https://github.com/bellafard/DAT-Decoding) were written in MATLAB (R2019b, MathWorks). Statistical analyses were performed using Prism (v10, GraphPad).

For manuscripts utilizing custom algorithms or software that are central to the research but not yet described in published literature, software must be made available to editors and reviewers. We strongly encourage code deposition in a community repository (e.g. GitHub). See the Nature Portfolio guidelines for submitting code & software for further information.

Data

Policy information about availability of data

All manuscripts must include a data availability statement. This statement should provide the following information, where applicable:

- Accession codes, unique identifiers, or web links for publicly available datasets
- A description of any restrictions on data availability
- For clinical datasets or third party data, please ensure that the statement adheres to our policy

All essential data for understanding the conclusions of the manuscript are presented in the main text and in Extended Data. Source data supporting the findings of the manuscript is included in the supplemental materials.

Field-specific reporting

Please select the one below that is the best fit for your research. If you are not sure, read the appropriate sections before making your selection.

☒ Life sciences ☐ Behavioural & social sciences ☐ Ecological, evolutionary & environmental sciences

For a reference copy of the document with all sections, see nature.com/documents/nr-reporting-summary-flat.pdf

Life sciences study design

All studies must disclose on these points even when the disclosure is negative.

Sample size	Sample sizes were selected to effectively capture experimental effects with the fewest animals, adhering to ethical standards and mirroring those in comparable studies (e.g., Nguyen et al. 2024, Nature; Vestergaard et al. 2023, Nature; Toader et al. 2023, Cell). They are considered adequate for the observed effects and consistency. Details are provided in the paper and Methods section.
Data exclusions	In optogenetic studies, animals were excluded if post-examination revealed incorrect viral injections or fiber placements. Exclusion criteria were set in advance.
Replication	All behavioral, imaging, and optogenetic experiments were replicated across several animals, yielding consistent results. See Methods for the exact number of animals and/or trials for each experiment.
Randomization	Animals were randomly allocated into the experimental groups. Trial types were pseudo randomly determined by a computer program in real time.
Blinding	Experimenters knew the group allocations during experiments and outcome evaluations but were blinded during recordings. Both control and experimental groups underwent identical conditions, except for specific treatments or manipulations. Data analysis for both groups followed the same criteria and methods.

Reporting for specific materials, systems and methods

We require information from authors about some types of materials, experimental systems and methods used in many studies. Here, indicate whether each material, system or method listed is relevant to your study. If you are not sure if a list item applies to your research, read the appropriate section before selecting a response.

Materials & experimental systems

n/a	Involved in the study	
☒	☐	Antibodies
☒	☐	Eukaryotic cell lines
☒	☐	Palaeontology and archaeology
☐	☒	Animals and other organisms
☒	☐	Human research participants
☒	☐	Clinical data
☒	☐	Dual use research of concern

Methods

n/a	Involved in the study	
☒	☐	ChIP-seq
☒	☐	Flow cytometry
☒	☐	MRI-based neuroimaging

Animals and other organisms

Policy information about studies involving animals; ARRIVE guidelines recommended for reporting animal research

Laboratory animals	All mice were 2 to 4-months old male and female C57BL/6J (Jackson Laboratory, Stock No: 000664) or C57BL/6J-Tg (Thy1-GCaMP6s) GP4.12Dkim/J (Jackson Laboratory, Stock No: 025776) or B6;DBA-Tg(tetO-GCaMP6s)2Niell/J (Jackson Laboratory, Stock No: 024742) crossed with B6.Cg-Tg(Camk2a-tTA)1Mmay/DboJ (Jackson Laboratory, Stock No: 007004). Animals were kept under a 12-hour light-

	dark cycle (lights on from 8 p.m. to 8 a.m.) with constant food access and underwent experiments during their dark cycle. They were water-restricted for a week before behavioral training began. See Methods for water restriction details.
Wild animals	No wild animals were used in the study.
Field-collected samples	No field collected samples were used in the study.
Ethics oversight	All experiments were conducted in accordance with National Institute of Health (NIH) guidelines and with the approval of the Chancellor's Animal Research Committee of the University of California, Los Angeles.

Note that full information on the approval of the study protocol must also be provided in the manuscript.
